# Susceptibility of *Aedes albopictus* and *Culex quinquefasciatus* to Japanese encephalitis virus

**DOI:** 10.1186/s13071-022-05329-0

**Published:** 2022-06-16

**Authors:** Luis M. Hernández-Triana, Arran J. Folly, Sanam Sewgobind, Fabian Z. X. Lean, Stuart Ackroyd, Alejandro Nuñez, Sarah Delacour, Andrea Drago, Patrizia Visentin, Karen L. Mansfield, Nicholas Johnson

**Affiliations:** 1 Vector Borne Diseases, Animal and Plant Health Agency, Addlestone, Surrey, UK; 2 Pathology and Animal Sciences Department, Animal and Plant Health Agency, Addlestone, Surrey, UK; 3 Veterinary Faculty, University of Zaragoza, Zaragoza, Spain; 4 Entostudio SrL, Viale del Lavoro 66, Ponte San Nicolò, Padua, Italy

**Keywords:** Mosquito, Invasive mosquitoes, Japanese encephalitis, Zoonosis, Vector competency, Emerging infectious disease

## Abstract

**Background:**

Japanese encephalitis virus (JEV) is the principal cause of mosquito-borne encephalitis in human populations within Asia. If introduced into new geographic areas, it could have further implications for public and animal health. However, potential mosquito vectors for virus transmission have not been fully investigated. The Asian tiger mosquito, *Aedes albopictus*, has emerged in Europe and is now expanding its geographical range into more northerly latitudes. *Culex quinquefasciatus*, although absent from Europe, has been detected in Turkey, a country with territory in Europe, and could act as a vector for JEV in other regions. To assess the risk of these invasive species acting as vectors for JEV and therefore potentially contributing to its geographical expansion, we have investigated the vector competence of *Ae. albopictus* and *Cx. quinquefasciatus*.

**Methods:**

Two colonised lines of *Ae. albopictus* (Italy and Spain) and a line of *Cx. quinquefasciatus* (Tanzania) were compared for susceptibility to infection by oral feeding with JEV strain SA-14, genotype III at 10^6^ PFU/ml and maintained at 25 °C. Specimens were processed at 7 and 14 days post-inoculation (dpi). Rates of infection, dissemination and transmission were assessed through detection of viral RNA by real-time polymerase chain reaction (RT-PCR) in mosquito body, legs and saliva, respectively, at each time point. Where possible, infection and dissemination were confirmed by immunohistochemical (IHC) detection of the JEV envelope protein.

**Results:**

*Aedes albopictus* from Italy showed no susceptibility to infection with JEV strain SA-14. Conversely, *Ae. albopictus* colonised in Spain was susceptible and 100% of infected mosquitoes that were subjected to saliva screening expressed viral RNA at 14 dpi. *Culex quinquefasciatus* was highly susceptible to infection as early as 7 dpi and 50% of infected mosquitoes that were subjected to saliva screening expressed viral RNA at 14 dpi. Infection and dissemination were confirmed in *Cx. quinquefasciatus* by IHC detection of JEV envelope protein in both the mid-gut and salivary glands.

**Conclusions:**

*Aedes albopictus* from two different locations in Europe range from being susceptible to JEV and capable of transmission through to being resistant. *Culex quinquefasciatus* also appears highly susceptible; therefore, both species could potentially act as vectors for JEV and facilitate the emergence of JEV into new regions.

**Graphical Abstract:**

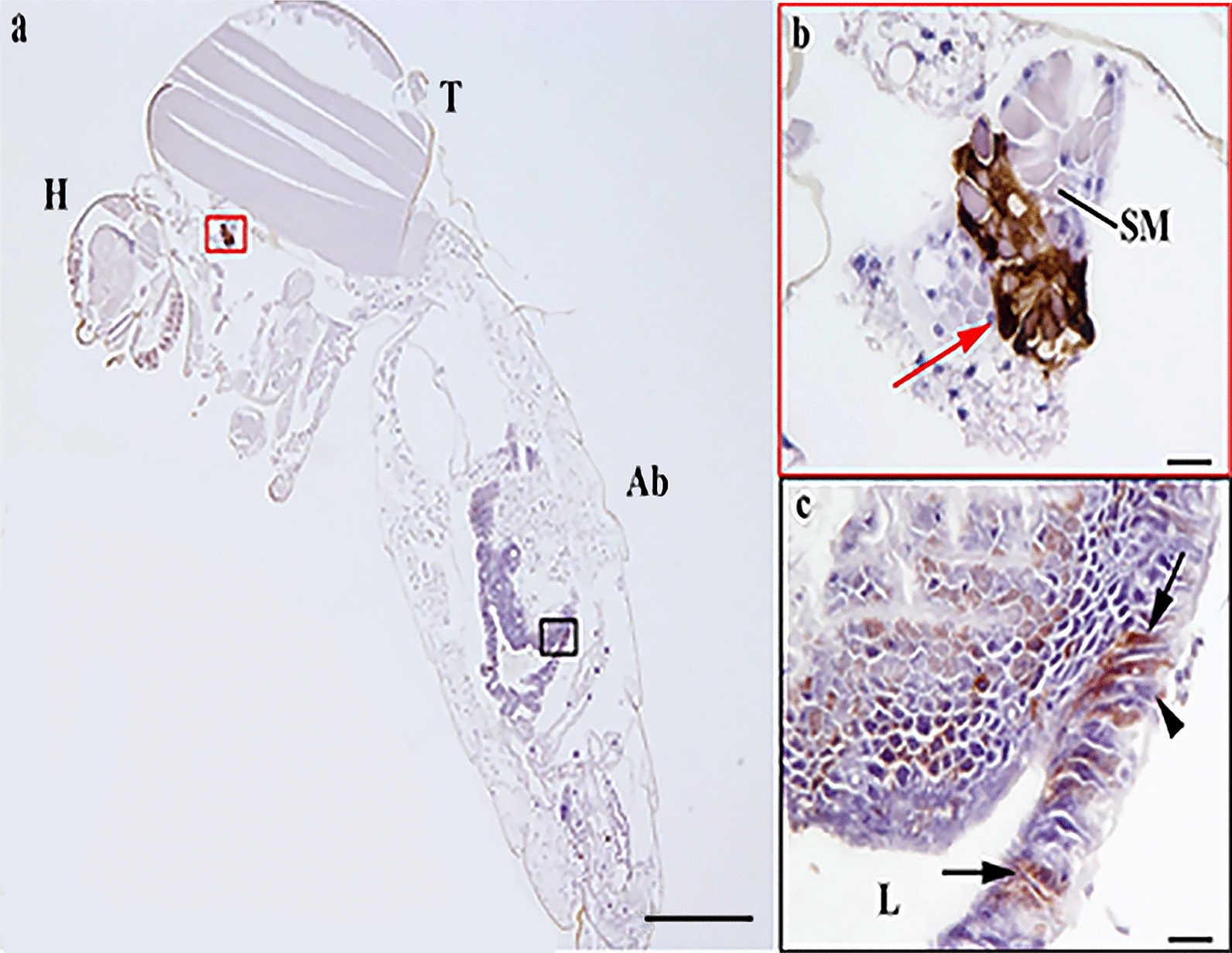

**Supplementary Information:**

The online version contains supplementary material available at 10.1186/s13071-022-05329-0.

## Background

Flaviviruses have a global distribution and many species can be transmitted by arthropods such mosquitoes, ticks and sandflies [1, 2]. Japanese encephalitis virus (JEV) (Family Flaviviridae, Genus *Flavivirus*) is the main aetiological agent for human viral encephalitis in the Far East and Southeast Asia. The World Health Organisation estimates that there are 68,000 clinical cases every year, but that over 3 billion people are at risk of exposure in Asia. Five genotypes are currently recognised, all endemic in Asia [3, 4].

Japanese encephalitis virus is maintained in an enzootic cycle between mosquito vectors and avian hosts, especially wading birds [5], although recent infection studies have demonstrated that domestic birds and pigs can act as amplifying hosts for JEV [6]. Some mosquito species can act as bridge vectors transmitting the virus to humans and livestock. Humans and other mammals such as horses are considered dead-end hosts due to low levels of viraemia, although recent studies have shown that pig-to-pig transmission can occur by the oronasal infection route [5, 7]. Due to a combination of climate change, movement of people and livestock, migratory birds, and the introduction of invasive vectors into new areas, geographic expansion of JEV into Europe could occur in the future [8, 9].

*Culex tritaeniorhynchus* is the main mosquito vector of JEV in regions where JEV is endemic, but other species, particularly within the genus *Culex,* can act as vectors [3, 10]. In Europe, *Cx. tritaeniorhynchus* has only been reported in Greece [11], and JEV RNA has been detected in a pool of *Cx. pipiens* in northern Italy [12], although this has not been associated with infection in either humans or swine. Recent experimental studies have demonstrated the competence of European populations of mosquitoes to transmit JEV, including *Cx. pipiens* from the UK and France, as well as *Culiseta annulata* and *Aedes detritus* from the UK [13–15]. Vector competence studies carried out by de Wispelaere et al*.* [16] demonstrated that a French population of the invasive species *Ae. albopictus* was vector competent for JEV genotypes III and V, and Jansen et al*.* [17] showed that infected *Ae. japonicus japonicus* from Germany expressed JEV in saliva at 14 days post-inoculation (dpi). However, there are many unassessed indigenous mosquito vectors with endemic European populations that may be able to transmit JEV and facilitate its emergence in the continent. In addition, non-native species to Europe have the potential to act as JEV vectors. These include *Cx. quinquefasciatus*, which has been found in Turkey [18]. Also, *Ae. albopictus* and *Ae. japonicus japonicus* are now distributed in several European countries [19, 20]. The detection of *Cx. quinquefasciatus* in Turkey could indicate that this mosquito species might expand its geographical range into Europe in the near future. Globally, different populations of *Ae. albopictus* have been identified as competent vectors of JEV, including Australia and Taiwan [21–23], and *Cx. quinquefasciatus* in North America [24], Brazil [13], India [25] and a colony from Queensland in Australia [26] were also competent to transmit JEV. However, other studies have shown that wild caught populations of *Cx. quinquefasciatus* from Australia and New Zealand were not competent to transmit JEV [26, 27], and two strains of *Ae. albopictus* (Yungho and Liyang, Taichung County) were less efficient vectors compared with a strain originating from Sanhsia (Taipei County) from Taiwan.

Due to the continued risk of invasive mosquito species globally, and the potential emergence of JEV into new areas [15], this study assessed the vector competence of two populations of *Ae. albopictus* (originating from Italy and Spain) and *Cx. quinquefasciatus* as a control for JEV genotype III. In this study, we selected to use JEV genotype III to inoculate mosquitoes as it is one of the most prevalent JEV genotypes along with genotype I, and it is associated with temperate climates [4]. In addition, immunohistochemistry (IHC) techniques were utilised to assess the presence of JEV antigen in mosquito histological sections, which facilitated the visualisation of JEV distribution within the context of specific mosquito tissues.

## Methods

### Colonisation of mosquitoes

Laboratory colonies comprised *Ae. albopictus* (Padua, Italy) (year of colonisation unknown and donated by Entostudio, Italy), *Ae. albopictus* (Barcelona, year of colonisation 2009 and donated by Universidad de Zaragoza, Spain) and *Cx. quinquefasciatus* (established at the Tropical Pesticides Research Institute (TPRI), Arusha, East Tanzania) (year of colonisation at London School of Hygiene and Tropical Medicine 2010 and donated by London School of Hygiene and Tropical Medicine, UK). A colonised line of *Cx. quinquefasciatus* originating from Africa was included for comparison as the species is known to be vector competent for JEV. Maintenance of *Culex* and *Aedes* mosquitoes in an insectary within biosecurity level 3 laboratories followed previously published protocols [19, 28].

### Virus stocks

Japanese encephalitis virus genotype III (strain SA-14, isolated from *Cx. pipiens* larvae, China 1954) was donated by Dr. Jonas Schmidt-Chanasit, Bernhard Nocht Institute for Tropical Medicine, Hamburg, Germany). Virus stocks were propagated in Vero cells as previously described [15, 19, 28]. Briefly, virus was propagated in Vero E6 cells in 25 ml of a culture medium consisting of Eagles minimal essential medium (E-MEM-Sigma Aldrich, UK), with 10% foetal bovine serum (FBS) and penicillin-streptomycin-nystatin solution (1% Thermo Fisher Scientific) at 37 °C and 5% CO_2_ for 3 days in T75 flasks. Infection of the cell monolayer was confirmed by light microscopy to assess the cytopathic effect (CPE).

### Assessment of vector competence

Adult females of *Ae. albopictus* (Italy and Spain) and *Cx. quinquefasciatus* (Tanzania) were tested for their vector competence for JEV genotype III at 25 °C, which is representative of peak summer temperatures in the UK. Mosquitoes were provided with an infectious blood meal, composed of defibrinated horse blood, adenosine 5′-triphosphate (final concentration 0.02 mM) and virus stock to give a final virus concentration of 1.8 × 10^6^ PFU/ml (plaque forming units), using a Hemotek membrane feeding system (Hemotek Ltd Accrington, Lancashire, UK). Five- to ten-day-old adult female mosquitoes of both species were first starved of sucrose for 5 h and then allowed to feed on the infectious blood meal (as described above) in Bugdorm insect cages of 22 × 22 × 22 cm (Bugzarre.co.uk, Suffolk, UK) from 16:00, for a minimum of 16 h. The following day they were anaesthetised with triethylamine (TEA) FlyNap® (Blades Biological Limited, Edenbridge, UK) and separated into groups of blood-fed and non-blood-fed specimens. Only blood-fed mosquitoes were used to assess vector competence. For the processing of specimens and assessment of vector competence (infection, dissemination and transmission rates), a modified protocol adapted from [15, 19, 28] was followed. The transmission efficiency (TE) was calculated only at dpi 14, and it was defined as the number of virus-positive saliva samples per total number of fed females. Briefly, at 7 and 14 dpi, mosquitoes were immobilised at − 80 °C for 2 min and held in a plastic pot embedded in ice to ensure that they remained immobile during processing. Legs and wings were removed, saliva samples taken and the bodies, legs and wings, and saliva retained at − 80 °C for downstream analysis.

### Processing of samples for molecular detection of JEV RNA

The protocol of [15] was used for detection of JEV RNA in tissues using previously published primers [29], which target and amplify a fragment of the NS1 gene. A sample was considered positive for JEV RNA at a cycle threshold (ct) value of 39 or lower, based on validation trials of the JEV PCR against positive and negative samples. Instead of using a standard to calculate RNA copies per mosquito, we opted to use ct values, which would enable direct comparison with previous studies [15].

### Immunohistochemistry

The presence of JEV antigen in mosquitoes was determined by immunohistochemistry (IHC) in histological sections. All segments of the mosquito, head, thorax and abdomen, were examined by light microscopy. A sufficient number of blood-fed specimens was available to assess by IHC for *Cx. quinquefasciatus* only. Unfortunately, fewer female *Ae. albopictus* took an infectious blood meal; therefore, the number of mosquitoes available for the vector competence experiment was lower, and we were not able to retain any specimens for IHC. Briefly, 12 infected blood-fed and 3 non-infected control female mosquitoes were placed in 10% neutral buffered formalin for fixation for 48 h. After fixation the wings and extremities were removed and the body was placed in sagittal plane prior to routine processing to paraffin blocks. Serial 3-μm-thick sections of the formalin-fixed paraffin-embedded (FFPE) mosquitoes were cut and placed on silane-coated slides (3-trietoxysilyl-propylamine). Proteinase enzyme buffer (DAKO, Ely, Cambridgeshire, UK) applied for 15 min at 20 °C was used as the antigen retrieval method. A mouse monoclonal anti-Flavivirus E-glycoprotein antibody (ab155882, Abcam, Cambridge, UK) applied at 1 in 50 dilution in Tris-buffered saline with 0.05% Tween 20 (TBST, VWR, Leicestershire, UK) at 4 °C for 18–20 h (overnight) was used as primary antibody to detect JEV. Parallel sections were tested with a protein concentration matched mouse immunoglobulin G class 2a (Abcam, Cambridge, UK) as isotype controls to identify any non-specific immunolabelling. Slides were then washed in purified water and assembled into coverplates for immunolabelling. DAKO mouse EnVision™ + System, HRP Peroxidase (DAKO, Ely, Cambridgeshire, UK) was used as a secondary antibody and incubated for 30 min at 20 °C combined with swine and goat immune serum (Vector Laboratories, Peterborough, UK). Antibody binding was visualised using the chromogen 3,3′-diaminobenzidine (DAB) + substrate-chromogen, which results in a brown-colored precipitate at the antigen site after 10-min incubation. Finally, sections were counterstained with Mayer’s haematoxylin (HE) and mounted in Distyrene Plasticiser Xylene (DPX) mounting medium (TCS Bioscience, Buckingham, UK) for light microscopy. Sections of West Nile virus-infected mouse brain and JEV-infected Vero cells were used as positive controls for flavivirus immunostaining.

### Virus titration

Titrations of both stock virus and virus in the infected blood meal were performed by plaque assay as previously described [19, 28].

### Statistical analysis

The graphical output was carried in the R programme (http://www.R-project.org). A *t-*test was performed comparing the ct values for RT-PCR to measure relative levels of virus infection between *Cx. quinquefasciatus* and *Ae. albopictus*.

## Results

A total of 81 females (28.9%) from *Ae. albopictus* originating from Italy successfully fed following an infectious blood meal containing JEV, while 106 females (52.7%) of *Ae. albopictus* originating from Spain fed on the bloodmeal (Table 1). Conversely, only 79 females (37.4%) of *Cx. quinquefasciatus* fed (Table 2). In general, mortality was observed between 1 and 2 dpi, then the survival of mosquitoes was relatively stable until 6 dpi before declining towards 13–14 dpi, which is typical for vector competence experiments (Additional file 1: Fig. S1).

**Table 1:** Infection, dissemination and transmission of *Aedes albopictus* (Italy and Spain) following consumption of a blood meal containing Japanese encephalitis virus genotype III

Species	Blood meal titre (PFU/ml)	Blood feeding rate (%)		Dpi 7 (%)^a^	Dpi 14 (%)^a^
*Aedes albopictus,* Padua, Italy	1.8 × 10^6^	81/281 (29)	Infection (body)	0/19 (0)	0/28 (0)
			Dissemination (legs)	0	0
			Transmission (saliva)	0	0
*Aedes albopictus,* Barcelona, Spain	1.8 × 10^6^	106/201 (53)	Infection (body)	1/18 (6)	4/24 (17)
			Dissemination (legs)	0/1 (0)	3/4 (75)
			Transmission (saliva)	0	3/3 (100)

Groups of mosquitoes were maintained at 25 °C for the indicated time periods

*Dpi* days post-inoculation

^a^Number of mosquitoes dissected is shown by the denominator

**Table 2:** Infection, dissemination and transmission of *Culex quinquefasciatus* following consumption of a blood meal containing Japanese encephalitis virus genotype III

Species	Blood meal titre (PFU/ml)	Blood feeding rate (%)		Dpi 7 (%)^a^	Dpi 14 (%)^a^
*Culex quinquefasciatus,* Tanzania	1.8 × 10^6^	79/211 (37)	Infection (body)	5/10 (50)	23/29 (79)
			Dissemination (legs)	1/5 (20)	4/23 (17)
			Transmission (saliva)	1/1 (100)	2/4 (50)

Groups of mosquitoes were maintained at 25 °C for the indicated time periods

*Dpi* days post-inoculation

^a^Number of mosquitoes dissected is shown by the denominator

Virus infection, dissemination and transmission were initially determined by RT-PCR. *Aedes albopictus* originating from Italy did not show evidence of infection, dissemination or transmission at either 7 or 14 dpi (Table 1). However, *Ae. albopictus* originating from Spain did show infection at 7 dpi (6%), but neither dissemination nor transmission was detected. However, at 14 dpi infection (17%), dissemination (75%) and transmission (100%, *n* = 3) were detected (Table 1). The overall transmission efficiency of *Ae. albopictus* from Spain at dpi 14 was 2.83%.

*Culex quinquefasciatus* showed higher prevalence of infection (50%), dissemination (20%) and transmission (100%, *n* = 1) at 7 dpi. In addition, higher prevalence of infection (79%), dissemination (17%) and transmission (50%, *n* = 2) were observed at 14 dpi (Table 2). The transmission efficiency of *Cx. quinquefasciatus* at dpi 14 was 2.53%.

**Fig. 1:**
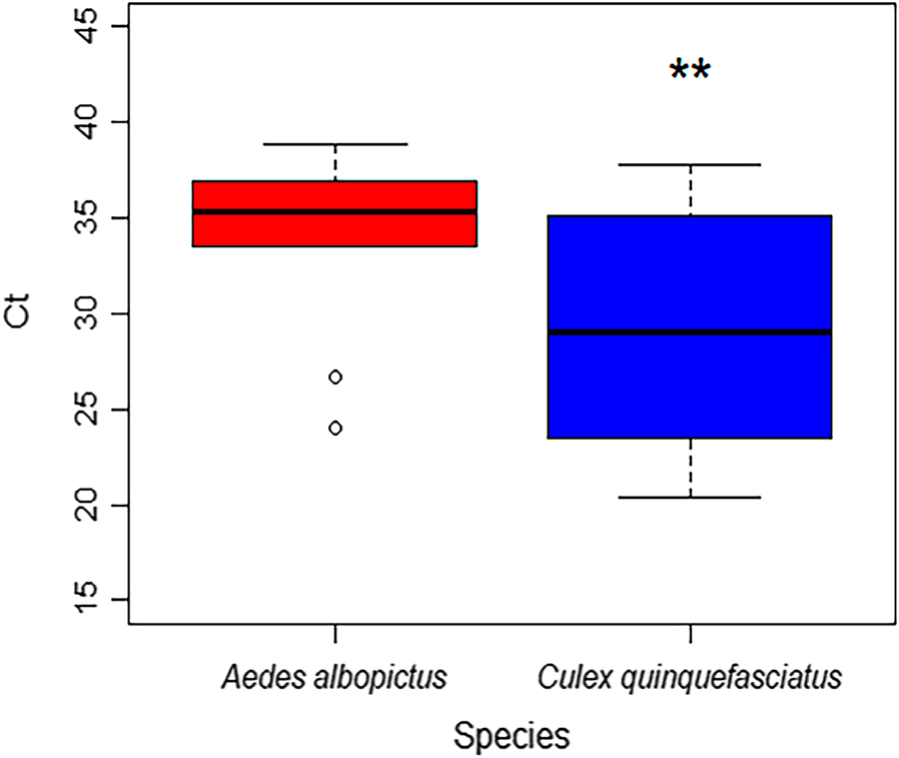
Boxplot comparing the cycle threshold (ct) values for *Ae. albopictus* and *Cx. quinquefasciatus* from RNA extractions of specimens infected with Japanese encephalitis virus and maintained at 25 °C. *Culex quinquefasciatus *ct values were significantly lower compared to *Ae. albopictus*, suggesting that quantity of viral RNA was higher in these samples. Significance (*P* < 0.05) denoted by a double asterisk (**)

To compare relative amounts of virus genome in the tissue we analysed, the threshold values from each amplification cycle (ct) were evaluated, in particular at 14 dpi (Fig. 1). The ct values detected from *Cx. quinquefasciatus* were significantly lower compared to *Ae. albopictus*, suggesting a higher level of viral RNA detection in these samples (*t* = 2163, *P* = 0.019).

**Fig. 2:**
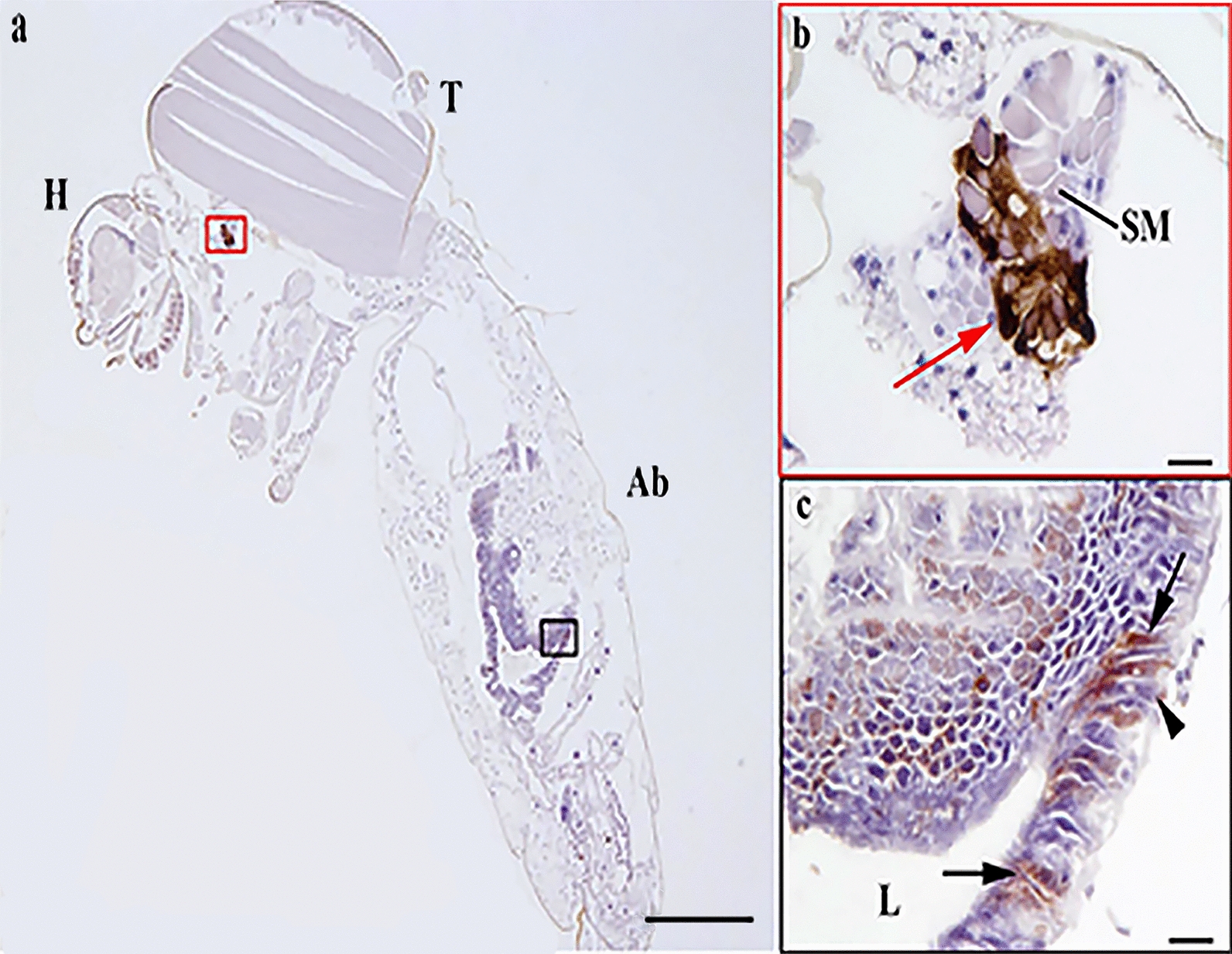
Japanese encephalitis virus infection at 25 °C of posterior midgut epithelial cells in *Cx. quinquefasciatus*. **a** Head (H), thorax (T), abdomen (Ab). **b** Intracytoplasmic immunolabelling in the distal lobes of salivary gland, defined by the presence of secretory masses (SM); intense antigen labelling particularly in the basal region of the epithelium (red arrow). **c** Antigen labelling in the apical ciliated cells (arrow) and basal epithelial cells (arrowhead) of the posterior midgut; lumen of the midgut (L). Scale bar: 500 µm (**a**); 20 µm (**b**, **c**)

Cellular distribution of JEV infection in *Cx. quinquefasciatus* was determined by IHC on sections of FFPE whole mosquitoes to detect JEV envelope antigen. JEV-immunolabeled cells were observed in the posterior midgut of seven infected females of *Cx. quinquefasciatus* (Fig. 2; Additional file 2: Fig. S2). Immunolabelling was present in clusters of epithelial cells, predominantly ciliated pseudostratified intestinal cells, located in the posterior midgut region, as characterised by dark brown pigment deposition within the cytoplasm. The levels of midgut epithelial cells infection were highly variable with labelling ranging from a continuous row of cells to single cells (Additional file 2: Fig. S2). Positive intracytoplasmatic immunolabelling was observed in the salivary gland of one specimen of *Cx. quinquefasciatus*, defined by the presence of secretory masses corroborating the detection of virus by this and other methods such as PCR (Table 2). Isotype control immunolabelled sections did not show any non-specific staining on infected mosquitoes.

## Discussion

Our results show that *Ae. albopictus* originating from Spain and a line of *Cx. quinquefasciatus* originating from Tanzania were susceptible to infection by JEV genotype III. In addition, our study demonstrates that after 14 days at 25 °C, JEV virus was able to disseminate throughout *Ae. albopictus* originating from Spain and *Cx. quinquefasciatus* with viral RNA being detected in saliva by RT-PCR, and also in the salivary glands of *Cx. quinquefasciatus* by IHC. This suggests that the studied populations may be competent vectors of JEV genotype III under our experimental conditions, corroborating previous findings from populations of *Ae. albopictus* and *Cx. quinquefasciatus* [13, 16, 21, 23, 24, 26, 30]. Of the mosquito populations assessed, *Cx. quinquefasciatus* appeared the more competent vector for JEV, as expected, demonstrating the highest levels of dissemination and transmission, despite blood feeding at a lower rate than *Ae. albopictus* from Spain. However, these results demonstrated that a population of *Ae. albopictus* originating from Spain may also act as a competent vector for JEV; this is also supported by the TE for JEV in both species.

Both *Ae. albopictus* and *Cx. quinquefasciatus* are indigenous in tropical areas, where temperatures > 30 °C can be encountered, and they are well known vectors for many arboviruses [24, 31–34]. Although *Cx. quinquefasciatus* has not yet been detected in Europe and its ability to successfully transmit JEV in Europe would require the establishment of high-density populations, its morphological, ecological and phylogenetic similarity to *Cx. pipiens* and its ability to colonise new areas via ship and airline vessels [35] make it a potentially important invasive species for studying the transmission of re-emerging zoonotic viruses such as JEV. The origin of *Cx. quinquefasciatus* is thought to be West Africa from where it colonised other regions through trade and migration. The species reached the Americas during the 1800s, spreading to Asia and the Pacific via whaling and merchant vessels [36, 37]. It is a common species in Africa; thus, our finding that the population from Tanzania is a highly competent vector for JEV is epidemiologically relevant in the event of JEV  spreading in the African continent.

Conversely, *Ae. albopictus* is now distributed in many countries in Europe and the Mediterranean Basin, with a few sporadic recorded incursions into more temperate regions such as the UK [38]. Several populations of *Ae. albopictus* are considered the main drivers for outbreaks of dengue and chikungunya fever in Europe [39]; it is also a known secondary vector of Zika virus in Latin America [28]. The species has spread rapidly throughout Europe, being mainly transported by road vehicles, where it can be considered a biting pest together with *Ae. japonicus japonicus* [40]. In our study, the Italian population of *Ae. albopictus* from Padua, Northern Italy, was not a competent vector for JEV genotype III, although it is an efficient vector of other arboviruses such as chikungunya virus [41]. Previous reports have suggested that the different origins of the Italian *Ae. albopictus* populations, which were introduced separately from different tropical and subtropical areas over the past 3 decades [41], could be the basis for differences in their vector competence. It is worth noting that the experimental conditions in this study maintained constant heat and humidity with a 24-h day-night photoperiod, which are standard conditions during vector competence studies [15, 19]. However, these conditions are not representative of natural conditions. Given that previous studies suggest that temperature appears to be a critical factor for both vector competence and vector mortality in this experimental system, we suggest that future studies incorporate variation between minimum and maximum temperature/humidity means to represent what occurs naturally [42]. Infection experiments carried out in *Cx. pipiens* have shown that a limiting factor at which this species becomes unable to transmit JEV genotype III is temperature, with higher temperatures (25 °C) causing increased mortality in infected mosquitoes compared to mosquitoes held at 20 °C [15]. However, no increased mortality was observed for *Ae. albopictus* or *Cx. quinquefasciatus* at 25 °C in the present study, suggesting that under our experimental conditions an elevated temperature and infection with JEV strain SA-14 did not cause additional mosquito mortality. This may be a consequence of the mosquito species and virus strain used as both naturally encounter higher temperatures than our experimental paradigm [3].

The labelling of virus antigen in *Cx. quinquefasciatus* confirmed that at 25 °C, JEV was able to infect the posterior midgut epithelial cells such as ciliated pseudostratified intestinal cells, which corroborates detection of virus in the mosquito body by molecular means. This supports a previous study showing that midgut epithelial cells are a major site of viral replication [15]. In addition, viral antigen was observed in mid-gut and salivary glands by IHC, which demonstrated that at 25 °C and by 14 dpi, the virus was able to overcome the midgut barrier and to infect secondary organs such as the salivary glands. Previous studies found that JEV present in the midgut appeared viable by the recovery of live virus in vitro from homogenised mosquito bodies [15]. However, it was unclear whether the restriction of JEV to the midgut was a result of active anti-viral control by the mosquitoes or the lower experimental temperature restricting virus replication. The authors suggested that an increase in temperature, or an increase in the duration of the experiment, could potentially trigger further virus replication and escape from the midgut; our results suggest that temperature may be a contributing factor to full viral dissemination.

## Conclusions

Of the mosquito populations studied, there was no evidence that the virus could infect or disseminate within the *Ae. albopictus* line originating from Italy at 25 °C at either 7 or 14 dpi. By contrast, *Ae. albopictus* originating from Spain and *Cx. quinquefasciatus* originating from Tanzania proved to be susceptible to infection as early as 7 dpi. Dissemination occurred in a proportion of infected mosquitoes and JEV was detected in the saliva of these mosquitoes. This suggests the potential of these mosquito populations to transmit JEV genotype III (strain SA-14). Considering that several mosquito species have been shown to be competent vectors for a number of arboviruses, our results contribute to this expanding dataset and indicate that if JEV were to emerge in new areas, there would be a number of mosquito populations that could facilitate its transmission and persistence.

## Supplementary Information

**Additional file 1: Figure S1.** Survival of *Ae. albopictus* (Italy and Spain) and *Cx. quinquefasciatus* (Tanzania) at 25 °C following a blood meal containing Japanese encephalitis genotype III over 14 days post-infection; DPI, days post-infection.

**Additional file 2: Figure S2.** Japanese encephalitis virus infection of the midgut in seven specimens of *Cx. quinquefasciatus* maintained at 25 °C*.* (a–e) Strong immunolabelling. (f, g) Moderate immunolabelling . Scale bar: 20 µm.

## Data Availability

All data generated by this study and used is presented within this published article.
