# Supplementary figures and images for: Susceptibility of Aedes albopictus and Culex quinquefasciatus to Japanese encephalitis virus
Source: Parasit Vectors. 2022 Jun 16;15:210. doi: 10.1186/s13071-022-05329-0 (PMC9204976; doi:10.1186/s13071-022-05329-0)

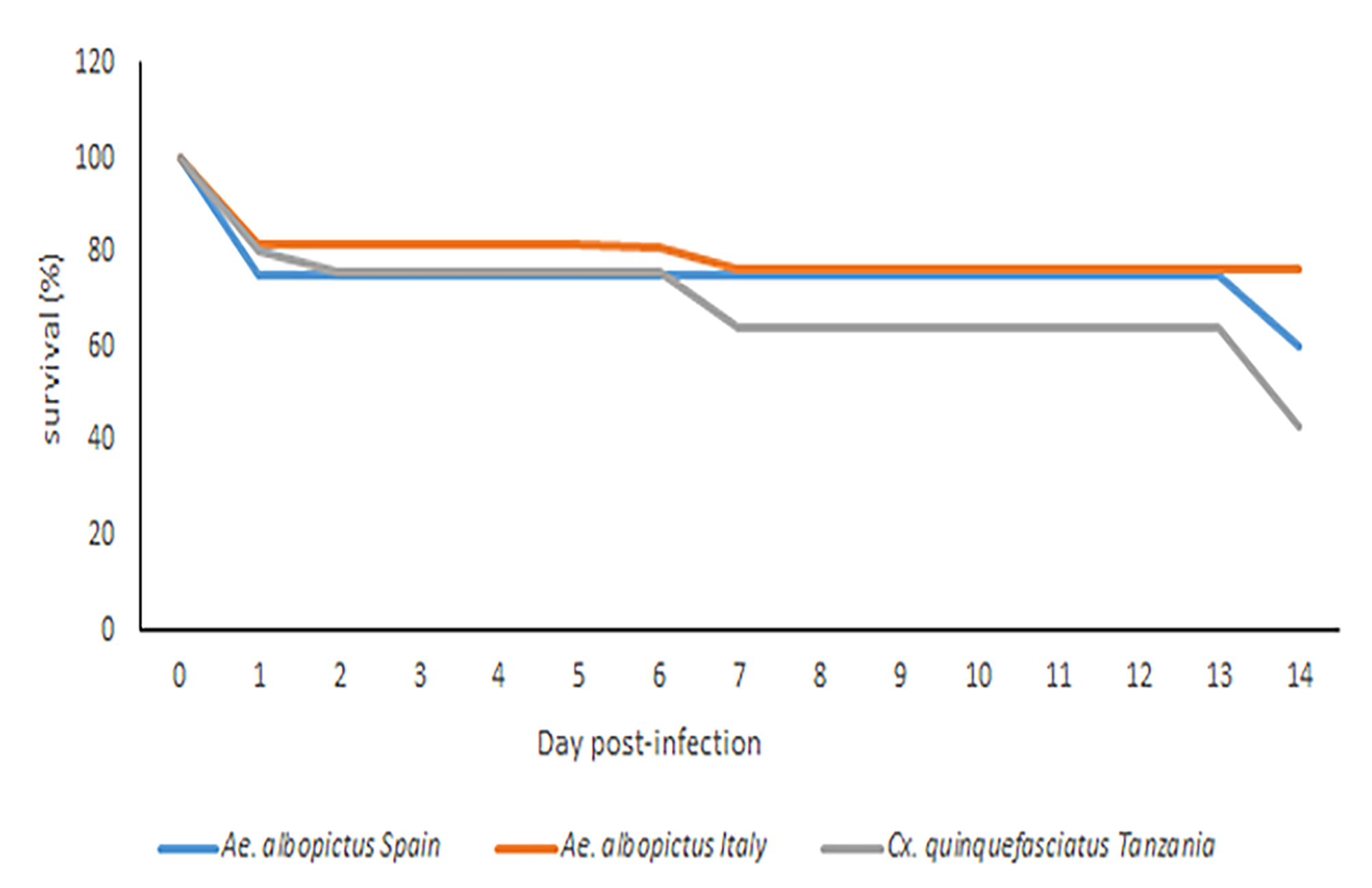

Supplement: Supplementary file 1 — Additional file 1: Figure S1. Survival of Ae. albopictus (Italy and Spain) and Cx. quinquefasciatus (Tanzania) at 25 °C following a blood meal containing Japanese encephalitis genotype III over 14 days post-infection; DPI, days post-infection. [file 13071_2022_5329_MOESM1_ESM.tif]

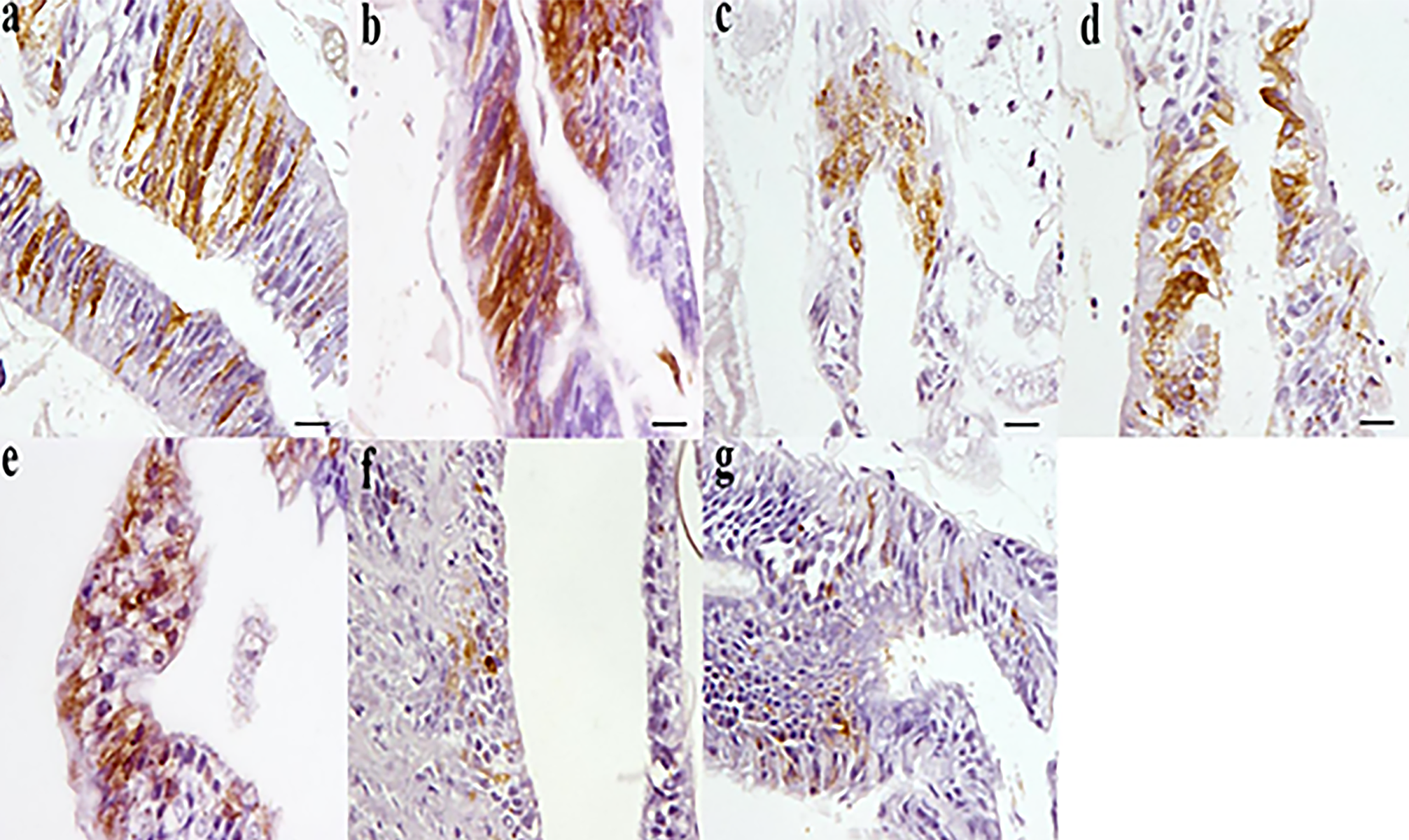

Supplement: Supplementary file 2 — Additional file 2: Figure S2. Japanese encephalitis virus infection of the midgut in seven specimens of Cx. quinquefasciatus maintained at 25 °C. (a–e) Strong immunolabelling. (f, g) Moderate immunolabelling . Scale bar: 20 µm. [file 13071_2022_5329_MOESM2_ESM.tif]
